# A large pedigree study confirmed the CGG repeat expansion of *RILPL1* Is associated with oculopharyngodistal myopathy

**DOI:** 10.1186/s12920-023-01586-9

**Published:** 2023-10-20

**Authors:** Xinzhuang Yang, Dingding Zhang, Si Shen, Pidong Li, Mengjie Li, Jingwen Niu, Dongrui Ma, Dan Xu, Shuangjie Li, Xueyu Guo, Zhen Wang, Yanhuan Zhao, Haitao Ren, Chao Ling, Yang Wang, Yu Fan, Jianxiong Shen, Yicheng Zhu, Depeng Wang, Liying Cui, Lin Chen, Changhe Shi, Yi Dai

**Affiliations:** 1 Department of Neurology, Peking Union Medical College Hospital, Chinese Academy of Medical Sciences & Peking Union Medical College, Beijing, 100730 People’s Republic of China; 2 Medical Research Center, State Key Laboratory of Complex Severe and Rare Diseases, Peking Union Medical College Hospital, Chinese Academy of Medical Sciences and Peking Union Medical College, Beijing, 100730 People’s Republic of China; 3 Department of Neurology, The First Affiliated Hospital of Zhengzhou University, Zhengzhou University, Zhengzhou, Henan 450000 People’s Republic of China; 4 GrandOmics Biosciences, Beijing, People’s Republic of China; 5 Department of Orthopaedic Surgery, Peking Union Medical College Hospital, Chinese Academy of Medical Sciences & Peking Union Medical College, Beijing, 100730 People’s Republic of China; 6 Laboratory of Clinical Genetics, Peking Union Medical College Hospital, Chinese Academy of Medical Sciences & Peking Union Medical College, Beijing, 100730 People’s Republic of China; 7 Academy of Medical Sciences of Zhengzhou University, Zhengzhou, Henan 450000 People’s Republic of China; 8 Henan Key Laboratory of Cerebrovascular Diseases, The First Affiliated Hospital of Zhengzhou University, Zhengzhou University, Zhengzhou, Henan 450000 People’s Republic of China; 9 Institute of Neuroscience, Zhengzhou University, Zhengzhou, Henan 450000 People’s Republic of China

**Keywords:** CGG repeat expansion, Long-read whole-genome sequencing, Neurodegenerative disease, Oculopharyngodistal myopathy, *RILPL1*

## Abstract

**Background:**

Oculopharyngodistal myopathy (OPDM) is an autosomal dominant adult-onset degenerative muscle disorder characterized by ptosis, ophthalmoplegia and weakness of the facial, pharyngeal and limb muscles. Trinucleotide repeat expansions in non-coding regions of *LRP12*, *G1PC1, NOTCH2NLC* and *RILPL1* were reported to be the etiologies for OPDM.

**Results:**

In this study, we performed long-read whole-genome sequencing in a large five-generation family of 156 individuals, including 21 patients diagnosed with typical OPDM. We identified CGG repeat expansions in 5’UTR of *RILPL1* gene in all patients we tested while no CGG expansion in unaffected family members. Repeat-primed PCR and fluorescence amplicon length analysis PCR were further confirmed the segregation of CGG expansions in other family members and 1000 normal Chinese controls. Methylation analysis indicated that methylation levels of the *RILPL1* gene were unaltered in OPDM patients, which was consistent with previous studies. Our findings provide evidence that *RILPL1* is associated OPDM in this large pedigree.

**Conclusions:**

Our results identified *RILPL1* is the associated the disease in this large pedigree.

**Supplementary Information:**

The online version contains supplementary material available at 10.1186/s12920-023-01586-9.

## Background

Oculopharyngodistal myopathies (OPDM) are a group of autosomal dominant, adult-onset neuromuscular diseases. The clinical features of OPDM are slowly progressive ptosis, ophthalmoplegia, facial and bulbar weakness and distal predominant limb muscle weakness and atrophy. The disease was first reported and nominated in 1977 [1], since then several hundred cases have been reported. The main diagnostic criteria are typical clinical manifestations, inheritance pattern and characteristic muscle biopsy findings including chronic myopathic change with rimmed vacuoles. With the development of long-read sequencing platform, four causative gene locations have been successfully discovered. OPDM type 1 (OPDM1; MIM 164310) [2] is caused by the expansion of CGG repeats in the 5’-untranslated region (5’UTR) of *LRP12* gene. OPDM type 2 (OPDM2; MIM 618940) [3] is due to the CGG repeat expansion in the 5’UTR of *GIPC1* gene. Meanwhile, the CGG repeat expansion in the 5’UTR of *NOTCH2NLC* gene is responsible for OPDM type 3 (OPDM3; MIM 619473) [4]. During the same period, two other Chinese team, Yu et.al. and Deng et al., found that CGG repeat expansion in the 5’UTR of *RILPL1* was also associated with OPDM patients (OMIM 619790) [5, 6], namely OPDM type 4.

The expansion of tandem repeat length causing human disorders affect the neurological system [7]. Autosomal-dominant familial adult myoclonic epilepsy (FAME) characterized by myoclonus and epilepsy is another example [8]. To date, at least the six genes harboring ATTTT/ATTTC expansion leading to FAME are confirmed [9–12]. These six genes have completely different functions and expression profiles. This fact strongly suggests that the pathogenicity of ATTTT/ATTTC repeats is independent of the recipient gene and its function, and that the gene is only a vessel for repeat expression. Similar to FAME, the three genes harboring CGG repeat expansions in 5’UTR have totally distinct functions and expression patterns. The CGG repeat expansion in *NOTCH2NLC* was initially recognized as the genetic cause for neuronal intranuclear hyaline inclusion disease and other neurodegenerative diseases affecting the central nervous system [2, 13–17].

In this study, we explored gene loci in a large Chinese pedigree with autosomal dominant OPDM. By using long-read whole-genome sequencing (LRS) on the Oxford Nanopore platform and PacBio SMRT platform, we found a CGG repeat expansion in the 5’UTR segment of *RILPL1* gene, was associated OPDM in this family.

## Methods

### Library preparation and whole-exome sequencing

Genomic DNA of peripheral blood leukocytes were obtained from all participants. The exome sequences were efficiently enriched from 0.4 μg genomic DNA using Agilent liquid capture system (Agilent SureSelect Human All Exon V6) according to the manufacturer’s protocol. DNA libraries were sequenced on Illumina NovaSeq for pair-end 150 bp reads. Valid sequencing data was mapped to the reference genome (GRCh37/hg19) by Burrows-Wheeler Aligner (BWA) software to get the original mapping results in BAM format. Subsequently, GATK (V3.8) were used to do variant calling and identify SNP and indels. Then, XHMM were used to identify CNVs. At last, ANNOVAR was performed to do annotation for VCF (Variant Call Format) file.

### Long-read whole-genome sequencing

DNA samples of affected individuals with OPDM and healthy individuals were sequenced using PromethION sequencer (Oxford Nanopore Technologies). Library preparation was carried out using a 1D Genomic DNA ligation kit (SQKLSK109) according to the manufacturer’s protocol. For each individual, one PRO-002 (R9.4.1) flowcell was used. Data analysis was followed by the pipeline in our previous work [18]. Briefly, PromethION data base-calling was performed using guppy v.3.3.0 (Oxford Nanopore Technologies), and only pass reads (qscore ≥ 7) were used for subsequent analysis. Long reads were aligned to reference genome (GRCh37/hg19) by minimap2 [19]. To validate the accuracy of ONT, we also performed PacBio Single Molecule, Real-Time (SMRT) DNA sequencing for patients FIII-39. HQRF (High quality Region Finder) were used to identify the longest region of singly-loaded enzyme activity. low-quality areas were filtered by Signal Noise Ratio (SNR). Subreads were obtained after the basic filtrations as previous reported. Circular Consensus Sequence (CCS) reads were retained using CCS tools and then aligned to the reference genome (GRCh37/hg19) using PBmm2.

### STR Detection and STR-Scoring Framework

The short tandem repeats that located in genic regions including 10 Kb up-/downstream of genes were identified in whole genome, using long-read whole-genome sequencing based on the STR-Scoring framework, as previously described [3]. Considering that nanopore reads have certain bias errors, we used IGV software [20] to further confirm and correct the repeat count of each read for the top 10 STRs, and refined the second largest repeat count as the estimated repeat count (ERC).

### 5mc DNA Methylation analysis

The Minimap2 [19] and Nanopolish [21] were applied for 5mC DNA methylation calling in ONT sequencing data. The methylation level around the CGG repeats and adjacent CpG island was compared between affected individuals and healthy individuals, and between expanded and non-expanded alleles using the Wilcoxon Rank Sum Test. Adjacent GpG was defined as chr12: 124,017,594–124,018,994, and CGG repeat region were defined as chr12:124,018,268–124,018,297 (GRCh37/hg19).

### RNA-seq analysis

Total RNAs were extracted from muscle tissues of patients III-42 and three controls following Trizol RNA isolation procedure. The quality of the input RNA was controlled using Agilent 2100. Total RNA samples were then applied to strand-specific, poly(A)-positive RNA-seq following the manufacturer’s protocols and pipelines as previously described [22, 23]. Deep sequencing was then performed on Illumina Hiseq X Ten sequencing systems with 151-bp paired-end reads mode. The RNA-seq achieved 14.98G ~ 16.54G data cross all samples. Raw reads were then aligned to human genome (GRCh37/hg19) by HISAT2 (V2.1.0) and read count were calculated by HTseq (V0.11.2). Gene expression level measured by RPKM were performed by our in-house Perl scripts [22].

### Identification of novel transcriptional start site of RILPL1

EST (Expressed Sequence Tag) data were obtained from UCSC (http://genome.ucsc.edu/, GRCh37/hg19) and CAGE-seq (Cap Analysis of Gene Expression AND deep Sequencing, BAM format) were download from FANTOM5 (https://fantom.gsc.riken.jp/5/) by searching for human skeletal muscle tissue data and we finally got three datasets with high data quality. BAM files were transformed into bigwig format and uploaded on UCSC browser for visualization.

### Repeat-Primed Polymerase Chain Reaction (RP-PCR)

RP-PCR were used for detecting CGG repeat expansions. The PCR mix with a total reaction volume of 10 μL was used, containing 0.25 U PrimeSTAR GXL DNA Polymerase, 1◊PrimeSTAR GXL Buffer, 200 μM each dATP, dTTP, dCTP (Takara Bio), and 7-deaza-dGTP (Sigma-Aldrich), 5% dimethyl sulfoxide (Sigma Aldrich), 1 M betaine (Sigma-Aldrich), 0.3 μM primer GENE-F and GENE-linker-R, 0.15 μM primer GENE- R, and 100 ng genomic DNA. The primers for all tested genes were listed in Supplementary Table 1. A slow-down PCR protocol was used. After incubation at 98 °C for 10 min, the cycling conditions were 98 °C for 30 s, 66 °C for 15 s with 1 °C reduction per cycle, and 72 °C for 4 min, for a total of 9 cycles. This was followed by 30 cycles of 98 °C for 30 s, 58 °C for 15 s, 72 °C for 4 min, and a final extension of 72 °C for 10 min. Electrophoresis was performed on a 3500xl Genetic analyzer (Thermo Fisher Scientific) and the data were analyzed using GeneMapper software (Thermo Fisher Scientific). The saw-tooth tail pattern in the electropherogram is suggestive of the disease-related repeat expansion.

### Fluorescence Amplicon Length Polymerase Chain Reaction (AL-PCR)

The PCR mix composition and thermal conditions in AL-PCR were almost the same as those for RP-PCR protocol, except for the use of different primer pairs RILPL1-AL-F and RILPL1-AL-R. The two primers for AL-PCR were RILPL1-AL-F: 5’-VIC-GCAACTCGGATCCCAACTTGG-3’ and RILPL1-AL-R: 5’-CAAACTCGTGCAACTCCCAAAC-3’. Electrophoresis was performed on a 3500xl Genetic analyzer with GeneScan 1200 LIZ dye Size Standard and the data was analyzed using GeneMapper software. The length of the highest signal peak of the expanded allele was used to calculate the number of repeats.

## Results

### Clinical and pathological features

**Fig. 1:**
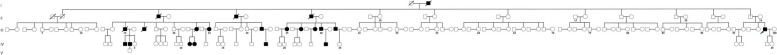
Pedigrees for the Chinese OPDM family presented in this study

**Fig. 2:**
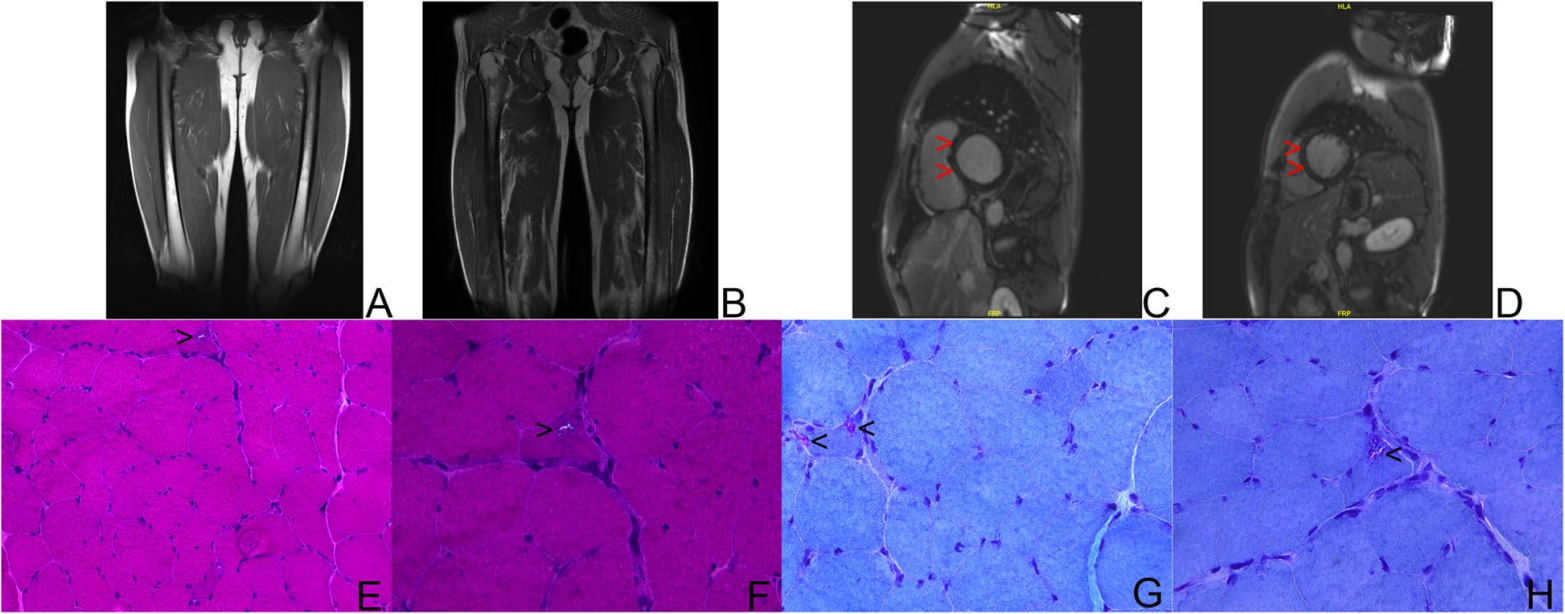
MR image and pathology of patient III-42. (A–B) T1-weighted MR images of thigh of index patient. **A**. image obtained in 2018; **B**. image obtained in 2021. Fatty infiltration in inner muscles was obvious in 2021, compared with almost no lesion in thigh muscles in 2018. **C**-**D** Gadolinium-enhanced cardiac MR imaging. Delayed myocardial enhancement was found in the middle of left ventricular wall (red arrow head). (**E**–**H** Skeletal muscle pathology in OPDM. **E**–F Haematoxylin and eosin staining showed mild variation in fiber size and rimmed vacuoles. (black arrow head), **E**. Magnification X20; **F**. magnification X20. **G**-**H** Modified Gomori Trichome staining demonstrated rimmed vacuoles. (black arrow head) G-H magnification X40

A large five-generation family of 156 individuals, including 21 patients diagnosed with OPDM were recruited from the neuromuscular clinic of Peking Union Medical College Hospital in China (Fig. 1). Patients with OPDM showed slowly progressive weakness in extraocular, laryngopharyngeal, facial, and upper and lower limb muscles. The weakness of limbs was distal predominance. In this family, the onset symptom was insidious, which was uniformly ptosis and ophthalmoparesis. The patients gradually developed dysphagia, dysarthria and limb weakness in the following years, and in the end stage, became bed-ridden. The intrafamily heterogeneity was obvious. Some patients remained stable in decades, while others experienced apparent progression in a few years. We followed up the index patient for more than 3 years. His main symptoms were limited to external ocular muscles with a course longer than 10 years. But the MR scan of his thigh muscles revealed a subclinical progressive fat infiltration in inner thigh muscles during a 3-year period (Fig. 2). The proband had complaints of palpitation and chest distress. Therefore, we performed gadolinium-enhanced cardiac MR imaging. We found delayed myocardial enhancement with a normal heart size and function. To the best of our knowledge, this is the first report of myocardial impairment in OPDM (Fig. 2). The pathological findings of muscle biopsy were in accordance with classical OPDM features. The histopathology of the quadriceps muscle showed mild myopathic changes with increase in fiber size and rimmed vacuoles in a few muscle cells (Fig. 2).

### Identification of CGG repeat expansions in RILPL1 gene in patients with OPDM

**Fig. 3:**
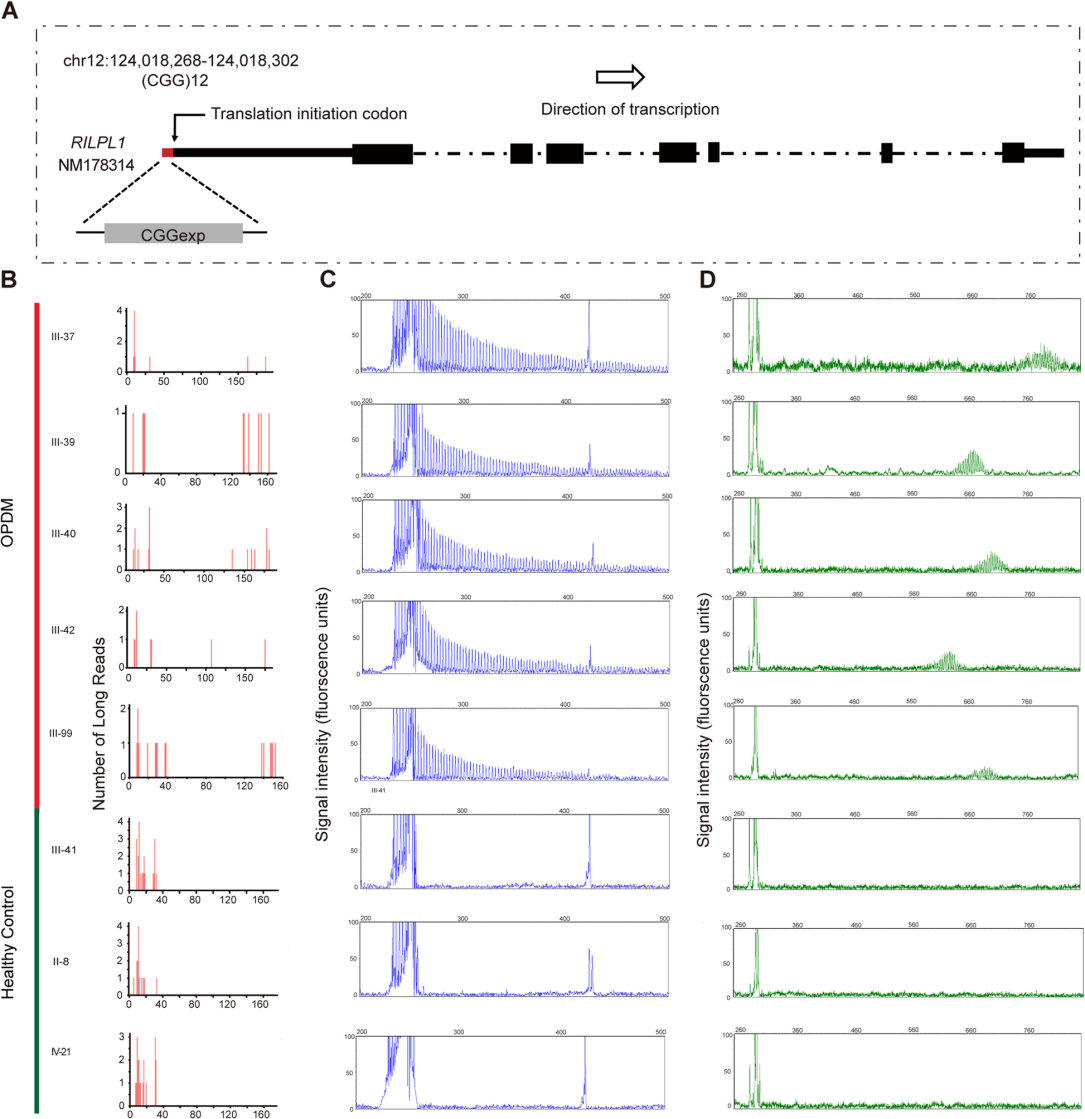
Identification and validation of the CGG repeat expansion in *RILPL1*. **A** Genomic position of the CGG repeat expansion in *RILPL1*. **B** Estimated repeat counts of the CGG expansion were more than 100 in five patients (III-37, III-39, III-40, III-42 and III-99) and only 30 in three healthy family members (II-8, III-41 and IV-21), as analyzed by long-read whole-genome ONT sequencing platform. **C** Representative results of RP-PCR analysis showing CGG repeat expansions of affected individuals while not in unaffected family members. **D** Representative results of AL-PCR analysis showing the numbers of expanded CGG repeats of OPDM individuals in family were ranging from 120 to 177. While in unaffected individuals, 9 units of CGG repeat were detected

To determine the genetic causes for patients in this family, we first checked the repeat expansion in *LRP12*, *GIPL1*, *NOTCH2NLC* and *PABPN1* which are pathogenic genes for OPDM and OPMD. However, they were all excluded by RP-PCR (Methods, Supplementary Fig. 1A-1H, Supplementary Table. 1). Considering the pathogenic mechanism for OPDM are all repeat expansions on genes’ 5’UTR according to previous reports, we performed long-read whole-genome sequencing by ONT PromethION sequencing platform on 5 affected (III-37, III-39, III-40, III-42 and III-99) and 3 unaffected family members (II-8, III-41 and IV-21) (Table 1) [24]. Gene coverage reached 98.73% in average, and mean depth is 14.77X (Supplementary Table. 2). Taking the advantage of long-read sequencing, repeats expansion exploration was performed by our previously reported STR-scoring method, which was a strategy to identify expanded repeats in the long reads based on comparisons between healthy and affected individuals (Methods) [3]. As results, 107, 287 short tandem repeats were identified in whole genome. Among the top10 candidate repeats, a heterozygous CGG repeat expansion in the upstream of *RILPL1* have a considerable high score (chr12:124, 018, 268–124, 018, 297, Fig. 3A, and Supplementary Table. 3). To avoid potential drawbacks of the pipeline which gave different weight factors to different gene regions and different patterns to the score, we re-evaluated the scores of those STRs without weight coefficients and confirmed that the CGG repeats in the upstream of *RILPL1* was still the most significant locus (Supplementary Table. 4). The repeat counts of this locus in 5 patients in this family were more than 100, while less than 40 copies in heathy individuals (Fig. 3B). To further confirm the result of ONT, we performed PacBio sequencing for patients III-39, which identified expanded CGG repeats in the same locus of *RILPL1* as with ONT data and demonstrated the uninterrupted CGG repeat expansions while not any other trinucleotide repeats types that are inserted in CGG consecutive sequence (Supplementary Fig. 2).

**Table 1:** Clinical features of affected individuals in the pedigree

*Patients*	III-25	III-32	III-35	III-37	III-39	III-40	III-42	III-99	IV-3	IV-4	IV-18	IV-24
Sex	F	M	F	F	F	M	M	M	M	M	M	M
Age at onset	39	37	38	40	29	35	28	21	25	29	26	21
Disease duration	21	8	15	12	20	12	13	11	16	10	4	2
Ptosis	+	+	+	+	+	+	+	+	+	+	+	+
Ophthalmoplegia	+	+	+	+	+	+	+	+	+	+	+	+
Bulbar weakness	+	+	+	-	+	+	-	+	+	+	-	-
Distal limb weakness	+	+	+	-	+	+	-	-	+	-	-	-
Proximal limb weakness	+	+	+	-	+	+	-	-	-	-	-	-
Creatine Kinase (U/L)	N/A	299	N/A	N/A	356	N/A	70	55	N/A	N/A	107	N/A
EMG pattern	N/A	Myopathic change	N/A	N/A	Myopathic change	N/A	N/A	Normal	N/A	N/A	N/A	N/A
CGG repeat number	160	N/A	N/A	177	135	145	120	144	137	127	130	N/A

No more samples were obtained from III-32, III-35, and IV-24 for AL-PCR

### RP-PCR and AL-PCR Analysis of Repeat Expansions in RILPL1

To further verify the CGG repeat expansions segregate with disease within family, RP-PCR were conducted surrounding CGG repeat expansions of *RILPL1*. RP-PCR analysis was first performed in family members which was supported by ONT sequencing data, and the CGG repeat expansions demonstrated in 5 affected members (III-37, III-39, III-40, III-42 and III-99) but not in 3 unaffected family members (II-8, III-41 and IV-21), which were perfectly consistent with the findings of ONT data (Figures. 3C and 3D). Subsequently, CGG repeat expansions were further examined in 12 additional family members, all 4 patients were confirmed CGG repeat expansions of *RILPL1* while not in other 8 unaffected family members (Supplementary Fig. 3A). We then determined the CGG repeat size using fluorescence amplicon length PCR (AL-PCR) analyses and found that all the affected individuals carried expanded CGG repeats, with the numbers of CGG repeat units ranging from 120 to 177 while 9 copies in all healthy family numbers (Table1, Fig. 3B). Besides, we performed PR-PCR and AL-PCR in 1,000 unaffected Chinese control subjects and showed 6 ~ 16 repeat units of CGG repeat in *RILPL1* (Supplementary Fig. 3B). All the above provided the evidence for the association between the CGG repeat expansion upstream *RILPL1* and OPDM in our study.

According to gene structures annotated by RefSeq, Ensembl and GENCODE, the CGG repeat is located 3 bp upstream of *RILPL1* gene (Methods, Fig. 3A, Supplementary Fig. 4). And, it is difficult to demonstrate the biological function if the CGG repeat doesn’t locate on gene body region. Interestingly, three independent pieces of evidence suggested the existence of an alternative transcription start site (TSS) upstream of CGG repeat (Supplementary Fig. 4). First, two RNA ESTs, including one spliced one, started from approximately 230 bp upstream of *RILPL1*. Second, consistent with EST evidence, CAGE-seq of skeletal muscles from FANTOM5 database also suggested an alternative TSS (Methods, Supplementary Fig. 4). Third, by analyzing our in-house RNA-seq data of skeletal muscles, we noted substantial RNA-seq signals upstream of the annotated TSS (Methods, Supplementary Fig. 4). In sum, we concluded that the transcription of *RILPL1* might initiate from an upstream TSS and include CGG repeats as part of its 5’UTR.

### Methylation Status and RNA level of RILPL1 in OPMD4

**Fig. 4:**
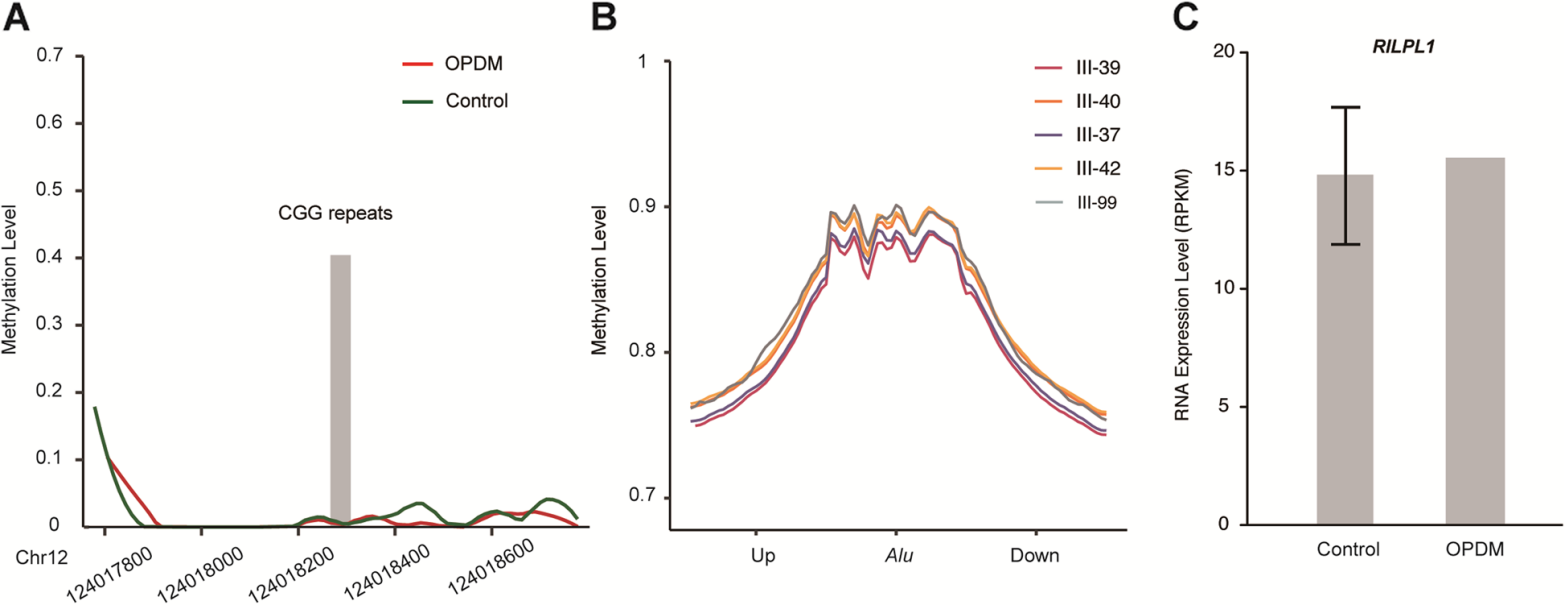
Methylation and RNA Expression of *RILPL1* gene. **A** Methylation status across the expanded CGG repeat region in *RILPL1* was determined using ONT data from five affected (III-37, III-39, III-40, III-42 and III-99) and three unaffected family members (II-8, III-41 and IV-21); no significant difference in methylation was detected between two groups. **B** Methylation status in *Alu* sequences along whole human genome, which are generally highly methylated in five RILPL1-affected individuals with OPDM, as positive controls. **C** RNA-seq analysis of *RILPL1* RNA expression levels in patient III-42 and three age-matched controls; no significant different was detected

To further evaluate the underlying mechanism of CGG repeat expansions in *RILPL1*, we detected 5mc (5-methylcytosine) DNA modification surrounding CGG repeats and adjacent CpG islands with ONT PromethION sequencing data (Methods). Consistent with other OPDM repeat-associated pathogenesis, there was no difference in DNA methylation levels between patients and healthy individuals[3] (Wilcoxon test, *P*-value = 0.30, Fig. 4A). As positive control, *Alu* repeat sequences were equally hypermethylated genome wide in both patients and controls, which confirmed that 5mc was accurately detected by nanopore sequencing (Fig. 4B). To further examine whether RNA level for *RILPL1* has changed within patients, we conducted RNA-sequencing with muscle tissues to analyze the transcription level. The results presented no significant difference between patient III-42 and age-matched controls (Methods, Fig. 4C, Supplementary Table. 5).

## Discussion

Oculopharyngodistal myopathy (OPDM) are a group of autosomal dominant neuromuscular diseases. The typical medical history is early-adult-onset ptosis and extraocular muscle palsy, followed by dysarthria, dysphagia and weakness of facial muscle, and later distal and proximal limb weakness. The disease is usually slowly progressive and the course always lasted for decades. The manifestations of systems other than skeletal muscle are rarely reported. In our large pedigree, except for early myocardial involvement of the proband suggested by the delayed enhancement in cardiac MR scan. The other patient, who was an elder cousin of the proband, was found to have severe iron deficiency anemia (Hb 58 g/L), which was treated with iron supplementation. No other systemic involvement was found in other patients of the pedigree.

Recently, four different gene loci were identified as causative pathogenesis for OPDM. The phenotypes of OPDM 1, 2, 3 and 4 are highly homogeneous [1], and the genotypes share obvious similarity. When we found this large multigenerational pedigree consistent with all the aspects of OPDM, we hope to find pathogenic genes of this family. Although traditional genetic linkage analysis could be in used in mapping the rough location of causative gene in this pedigree, we did not find the causative gene after genome-wide linkage analysis with WES data due to the family members available with WES were too far from each other and the statistical power was inadequate. With the development of long-read sequencing platform and advanced bioinformatic methods, we directly found a significant signal of a CGG repeat expansion in the upstream region of *RILPL1* using the STR-Scoring pipeline [3]. This expansion presented clear genotype and phenotype segregation in the family. It is worth mentioning that, another two back-to-back studies also confirmed this pathogenic gene in other pedigrees [5, 6].

*RILPL1*, Rab Interacting Lysosomal Protein Like 1, which was highly expressed in muscle tissues (Supplementary Fig. 5), plays a role in the regulation of cell shape and cellular protein transport. It is also a neuroprotective protein, involved in the regulation of neuron death by sequestering GAPDH in the cytosol and preventing the apoptotic function of GAPDH in the nucleus. *RILPL1* was reported to be associated with several neurological diseases, such as epilepsy and familial temporal lobe dementia, while not any description with OPMD.

Previous gene annotations showed that the CGG repeats were located in the upstream region of the transcription start site of *RILPL1* gene, which was 3 bp away from TSS. However, our analysis revealed that there was a novel TSS in the upstream region of *RILPL1*. We first found that there were ESTs, supporting the existence of RNA expression beyond the traditional transcription start site. To further confirm this was a novel TSS, we downloaded CAGE data of skeletal muscle tissues from FANTOM5 database and found that *RILPL1* had another significant CAGE peak in front of the annotated TSS, suggesting the existence of a new TSS. In addition, our analysis of in-house skeletal muscle RNA-seq data also confirmed the existence of RNA expression in the upstream of traditional TSS. Therefore, we proposed that there was a novel TSS and the CGG repeats were located in the 5’UTR of *RILPL1* gene.

In order to explore the biological effects of CGG repeat expansions, we first analyzed the DNA methylation level of *RILPL1*. No significant differences in methylation were found between patients and healthy members, which was consistent with previous reports. We also conducted methylation analysis for two alleles of heterozygotes and found no significant difference between them. It might suggest that CGG amplification may not work on methylation level. We subsequently explored gene expression differences with muscle RNA-seq data among patients and normal controls, and still found no significance on *RILPL1* expression between the two groups. We are now developing approach to convert iPSC (induced pluripotent stem cell) into skeletal muscle tissues to further investigate the underlying molecular mechanisms of OPDM4.

All the patients in this family followed the same pattern of disease progression. The onset symptom was exclusively ptosis and then other extraocular muscles, bulbar, distal and proximal limb muscles were sequentially affected. But the progression rate was apparently different. Some patients developed severe limb weakness in less than five years while others only had weakness and atrophy of ocular and pharyngolaryngeal muscle. Besides, we performed the correlation analysis between the number of repeats and the age at onset (r = 0.42, *P*-value = 0.26, Spearman correlation, Supplementary Fig. 6). The result was similar with the analysis conducted by Yu et.al [5]. The enhanced MR scan of index patient’s heart showed delayed myocardial enhancement in the left ventricular wall. This phenomenon pointed out that the myocardium was involved in OPDM.

In summary, we found that a CGG repeat expansion in *RILPL1* was responsible for OPDM in a large Chinese family. Our study widens the causative genes and loci spectrum of Oculopharyngodistal myopathy.

## Conclusion

Oculopharyngodistal myopathy (OPDM) is an autosomal dominant adult-onset degenerative muscle disorder characterized by ptosis, ophthalmoplegia and weakness of the facial, pharyngeal and limb muscles. In this study, we confirmed the pathogenic of CGG repeat expansions in 5’UTR of *RILPL1* gene in a large five-generation family of 156 individuals, including 21 patients diagnosed with typical OPDM.

### Supplementary Information

**Additional file 1: Supplementary Table 1.** Primer sequences for PCR. **Supplementary Table 2.** Statistics of long-read whole-genome sequencing for participants in this study. **Supplementary Table 3.** Top10 short tandem repeats with highest score identified by ONT sequencing data. **Supplementary Table 4.** Top10 short tandem repeats with highest score identified by ONT sequencing data. **Supplementary Table 5.** The characteristics of controls who contributed muscle tissue. Supplementary Figure 1. No repeat expansions in *PABPN1, LRP12*,* GIPC1 *and *NOTCH2NLC* were detected in patient III-39.No repeat expansions in *PABPN1* were detected with PCR and Sanger sequencing.No repeat expansions in *LRP12 *were detected by PCR and RP-PCR.No repeat expansions in *GIPC1* were detected by PCR and RP-PCR.No repeat expansions in *NOTCH2NLC *were detected by PCR and RP-PCR. **Supplementary Figure 2.** The CGG repeat expansion in the 5’UTR of *RILPL1* identified by PacBio CCS reads was consistent with ONTand uninterrupted CGG repeat expansions while no other repeat form was detected. **Supplementary Figure 3.** RP-PCR and AL-PCR analysis for additional family membersand frequency distribution of CGG repeat units in *RILPL1* among 1,000 healthy controls.** Supplementary Figure 4.** Gene annotation track from NCBI, UCSC, and GENCODE present traditional gene structures of *RILPL1*. While RNA ESTs, RNA-seq, and CAGE-seq provided evidences that *RIPL1* gene transcription might initiate from an upstream TSS and include expanded CGG repeats as part of its 5’UTR. **Supplementary Figure 5.** RNA expression level from Consensus dataset, HPA dataset, and GTEx dataset show that *RILPL1* is highly expressed in muscle and brain tissues.

## Data Availability

The raw sequence data reported in this paper have been deposited in the Genome Sequence Archive in National Genomics Data Center, China National Center for Bioinformation / Beijing Institute of Genomics, Chinese Academy of Sciences. The data (HRA002619, https://ngdc.cncb.ac.cn/gsa-human/browse/HRA002619) that support the findings of this study are available on request from the corresponding author. The data are not publicly available due to the patient consent requirements.
