# Supplementary material for: A large pedigree study confirmed the CGG repeat expansion of RILPL1 Is associated with oculopharyngodistal myopathy
Source: BMC Med Genomics. 2023 Oct 20;16:253. doi: 10.1186/s12920-023-01586-9 (PMC10590002; doi:10.1186/s12920-023-01586-9)
Supplement: Supplementary file 1 — Additional file 1: Supplementary Table 1. Primer sequences for PCR. Supplementary Table 2. Statistics of long-read whole-genome sequencing for participants in this study. Supplementary Table 3. Top10 short tandem repeats with highest score identified by ONT sequencing data. Supplementary Table 4. Top10 short tandem repeats with highest score identified by ONT sequencing data. Supplementary Table 5. The characteristics of controls who contributed muscle tissue. Supplementary Figure 1. No repeat expansions in PABPN1, LRP12, GIPC1 and NOTCH2NLC were detected in patient III-39.No repeat expansions in PABPN1 were detected with PCR and Sanger sequencing.No repeat expansions in LRP12 were detected by PCR and RP-PCR.No repeat expansions in GIPC1 were detected by PCR and RP-PCR.No repeat expansions in NOTCH2NLC were detected by PCR and RP-PCR. Supplementary Figure 2. The CGG repeat expansion in the 5’UTR of RILPL1 identified by PacBio CCS reads was consistent with ONTand uninterrupted CGG repeat expansions while no other repeat form was detected. Supplementary Figure 3. RP-PCR and AL-PCR analysis for additional family membersand frequency distribution of CGG repeat units in RILPL1 among 1,000 healthy controls. Supplementary Figure 4. Gene annotation track from NCBI, UCSC, and GENCODE present traditional gene structures of RILPL1. While RNA ESTs, RNA-seq, and CAGE-seq provided evidences that RIPL1 gene transcription might initiate from an upstream TSS and include expanded CGG repeats as part of its 5’UTR. Supplementary Figure 5. RNA expression level from Consensus dataset, HPA dataset, and GTEx dataset show that RILPL1 is highly expressed in muscle and brain tissues. [file 12920_2023_1586_MOESM1_ESM.docx]

**Supplementary Material**

Supplementary Table 1. Primer sequences for PCR.

| OPDM type | Gene | Method | Primer name | Primer sequence |
| --- | --- | --- | --- | --- |
| OPDM1 | LRP12 | RP-PCR | LRP12-F | 5'-FAM-GGAGGGAGGAGAAGCTGGAGGTAGACG-3' |
| OPDM1 | LRP12 | RP-PCR | LRP12-R | 5'-TACGCATCCCAGTTTGAGACGCGGCGGCGGCGGCGG-3' |
| OPDM1 | LRP12 | RP-PCR | LRP12-linker-R | 5'-TACGCATCCCAGTTTGAGACG-3' |
| OPDM1 | LRP12 | AL-PCR | LRP12-AL-F | 5'-FAM-GGGAGGAGAAGCTGGAGGTA-3' |
| OPDM1 | LRP12 | AL-PCR | LRP12-AL-R | 5'-GGAGAGCGCAGGGAGCAG-3' |
| OPDM2 | GIPC1 | RP-PCR | GIPC1-F | 5'-FAM-CAGACACATCCTTCTCGCAGAGGCCAC-3' |
| OPDM2 | GIPC1 | RP-PCR | GIPC1-R | 5'-TACGCATCCCAGTTTGAGACGCGGCGGCGGCGG-3' |
| OPDM2 | GIPC1 | RP-PCR | GIPC1-linker-R | 5'-TACGCATCCCAGTTTGAGACG-3' |
| OPDM2 | GIPC1 | AL-PCR | GIPC1-AL-F | 5'-FAM-CACATCCTTCTCGCAGAGGCCAC-3' |
| OPDM2 | GIPC1 | AL-PCR | GIPC1-AL-R | 5'-GAAGACGCGGATTGGCTGCGAGC-3' |
| OPDM3 | NOTCH2NLC | RP-PCR | NOTCH2NLC-F | 5'-FAM-GGCATTTGCGCCTGTGCTTCGGACCGT-3' |
| OPDM3 | NOTCH2NLC | RP-PCR | NOTCH2NLC-R | 5'-TACGCATCCCAGTTTGAGACGTCCTCCGCCGCCGCCGCC-3' |
| OPDM3 | NOTCH2NLC | RP-PCR | NOTCH2NLC-linker-R | 5'-TACGCATCCCAGTTTGAGACG-3' |
| OPDM3 | NOTCH2NLC | AL-PCR | NOTCH2NLC-AL-F | 5'-FAM-GGCATTTGCGCCTGTGCTTCGGACCGT-3' |
| OPDM3 | NOTCH2NLC | AL-PCR | NOTCH2NLC-AL-R | 5'-CACAGCAGAGCGGCGCAG-3' |
| OPDM4 | RILPL1 | RP-PCR | RILPL1-F | 5’-FAM-GCAACTCCCAAACTTGCCGCTGTCGAG-3’ |
| OPDM4 | RILPL1 | RP-PCR | RILPL1-R | 5’-CAGGAAACAGCTATGACCCCCGCTGCCGCCGCCGCC-3’ |
| OPDM4 | RILPL1 | RP-PCR | RILPL1-linker-R | 5'-CAGGAAACAGCTATGACC-3’ |
| OPDM4 | RILPL1 | AL-PCR | RILPL1-AL-F | 5'-VIC-GCAACTCGGATCCCAACTTGG-3' |
| OPDM4 | RILPL1 | AL-PCR | RILPL1-AL-R | 5’-CAAACTCGTGCAACTCCCAAAC-3’ |
| OPMD | PABPN1 | PCR | PABPN1-F | 5'-CGCAGTGCCCCGCCTTAGA-3' |
| OPMD | PABPN1 | PCR | PABPN1-R | 5'-ACAAGATGGCGCCGCCGCCCCGGC-3' |

Supplementary Table 2. Statistics of long-read whole-genome sequencing for participants in this study.

| Platform | ID | Patient/  healthy | base_number (M) | read_number (M) | Mean_base quality | mean_length (bp) | max_length (bp) | Mapped  rate | Mapped  depth |
| --- | --- | --- | --- | --- | --- | --- | --- | --- | --- |
| ONT | III-42 | patient | 51330.91 | 2.84 | 9.97 | 19,387 | 320,803 | 96.25 | 13.83 |
|  | III-40 | patient | 52037.26 | 2.68 | 9.87 | 20,687 | 266,190 | 96.57 | 14.11 |
|  | III-39 | patient | 61608.05 | 8.07 | 9.98 | 8,161 | 203,168 | 95.61 | 14.16 |
|  | III-37 | patient | 45595.29 | 6.64 | 9.56 | 7,137 | 567,325 | 95.62 | 12.12 |
|  | III-99 | patient | 62804.78 | 3.05 | 9.56 | 21,478 | 218,880 | 95.98 | 17.01 |
|  | IV-21 | healthy | 73112.22 | 9.88 | 8.99 | 7,834 | 278,441 | 94.92 | 16.04 |
|  | II-8 | healthy | 68770.20 | 6.73 | 8.98 | 10,734 | 263,203 | 95.42 | 15.25 |
|  | III-41 | healthy | 56341.24 | 3.22 | 9.89 | 18,476 | 268,066 | 96.51 | 15.66 |
| PacBio | III-39 | patient | 33560.83 | 2.18 | 27.54 | 15,364 | 54,527 | 99.15 | 10.83 |

Supplementary Table 3. Top10 short tandem repeats with highest score identified by ONT sequencing data (weighted score).

| **STR_ID** | **Pattern** | **Location** | **Gene** | **Patients** | | | | | **Healthy control** | | | **Healthy**  **Individuals**  **ERC Range** | **Healthy**  **Individuals**  **Cubic Mean** | **Score**  **（weighted）** |
| --- | --- | --- | --- | --- | --- | --- | --- | --- | --- | --- | --- | --- | --- | --- |
|  |  |  |  | **III-37** | **III-39** | **III-40** | **III-42** | **III-99** | **IV-21** | **II-8** | **III-41** |  |  |  |
| STR024749 | CGG | UTR5 | *RILPL1* | 187/0.00 | 171/0.00 | 178/0.00 | 107/0.00 | 154/0.00 | 32/0.70 | 31/0.95 | 33/0.32 | 13-38 | 32.298 | 2.61 |
| STR048412 | TCCC | UTR5 | *PTGIR* | 12/0.95 | 204/0.00 | 18/0.02 | 12/0.95 | 13/0.72 | 15/0.27 | 15/0.27 | 15/0.27 | 11-27 | 13.953 | 0.42 |
| STR099804 | CGGGG | UTR5 | *TDRD7* | 13/0.32 | 9/1.00 | 9/1.00 | 11/0.56 | 13/0.32 | 152/0.00 | 10/0.90 | 10/0.91 | 9-18 | 11.723 | 0.27 |
| STR036519 | TTA | UTR3 | *NKD1* | 24/0.09 | 27/0.01 | 18/0.99 | 19/0.84 | 18/0.99 | 19/0.84 | 593/0.00 | 18/0.99 | 17-38 | 20.595 | 0.16 |
| STR106369 | CCG | UTR5 | *PNMA3* | 19/0.12 | 10/0.62 | 11/0.47 | 10/0.62 | 11/0.47 | 9/0.78 | 29/0.01 | 29/0.01 | 7-29 | 13.228 | 0.15 |
| STR106035 | CGG | UTR5 | *HTATSF1* | 17/0.04 | 11/0.73 | 17/0.04 | 12/0.33 | 10/0.99 | 11/0.73 | 30/0.00 | 12/0.33 | 7-19 | 12.047 | 0.15 |
| STR033838 | GAAA | ncRNA_intronic | *NTRK3-AS1* | 102/0.01 | 102/0.01 | 67/0.07 | 88/0.03 | 63/0.15 | 72/0.03 | 87/0.03 | 66/0.08 | 1-68 | 51.071 | 0.13 |
| STR090145 | TCCC | downstream | *MYL10* | 7/1.00 | 8/0.88 | 8/0.88 | 9/0.68 | 9/0.68 | 229/0.00 | 8/0.88 | 13/0.05 | 7-34 | 10.868 | 0.12 |
| STR085352 | GGGA | upstream | *ABRACL* | 8/0.77 | 10/0.13 | 8/0.77 | 375/0.00 | 8/0.77 | 8/0.77 | 8/0.77 | 8/0.77 | 6-20 | 9.417 | 0.11 |
| STR097731 | CCG | UTR5 | *ERMP1* | 28/0.06 | 29/0.03 | 11/0.73 | 28/0.06 | 27/0.07 | 10/0.99 | 11/0.73 | 16/0.19 | 9-36 | 15.336 | 0.11 |

Supplementary Table 4. Top10 short tandem repeats with highest score identified by ONT sequencing data (unweighted score).

| **STR_ID** | **Pattern** | **Location** | **Gene** | **Patients** | | | | | **Healthy control** | | | **Healthy Individuals ERC Range** | **Healthy Individuals Cubic Mean** | **Score**  **(unweighted)** |
| --- | --- | --- | --- | --- | --- | --- | --- | --- | --- | --- | --- | --- | --- | --- |
|  |  |  |  | **III-37** | **III-39** | **III-40** | **III-42** | **III-99** | **IV-21** | **II-8** | **III-41** |  |  |  |
| STR024749 | CGG | UTR5 | *RILPL1* | 187/0.00 | 171/0.00 | 178/0.00 | 107/0.00 | 154/0.00 | 32/0.70 | 31/0.95 | 33/0.32 | 13-38 | 32.298 | 0.74 |
| STR036519 | TTA | UTR3 | *NKD1* | 24/0.09 | 27/0.01 | 18/0.99 | 19/0.84 | 18/0.99 | 19/0.84 | 593/0.00 | 18/0.99 | 17-38 | 20.595 | 0.16 |
| STR033838 | GAAA | ncRNA intronic | *NTRK3-AS1* | 102/0.01 | 102/0.01 | 67/0.07 | 88/0.03 | 63/0.15 | 72/0.03 | 87/0.03 | 66/0.08 | 1-68 | 51.071 | 0.13 |
| STR090145 | TCCC | downstream | *MYL10* | 7/1.00 | 8/0.88 | 8/0.88 | 9/0.68 | 9/0.68 | 229/0.00 | 8/0.88 | 13/0.05 | 7-34 | 10.868 | 0.12 |
| STR085352 | GGGA | upstream | *ABRACL* | 8/0.77 | 10/0.13 | 8/0.77 | 375/0.00 | 8/0.77 | 8/0.77 | 8/0.77 | 8/0.77 | 6-20 | 9.417 | 0.11 |
| STR038932 | GAAA | intronic | *CFAP52* | 102/0.01 | 95/0.01 | 46/0.17 | 102/0.01 | 67/0.09 | 38/0.67 | 48/0.15 | 93/0.01 | 32-107 | 48.362 | 0.10 |
| STR069005 | GAAA | ncRNA intronic | *IL12A-AS1* | 110/0.01 | 120/0.00 | 69/0.16 | 50/0.26 | 102/0.00 | 72/0.15 | 110/0.01 | 64/0.16 | 1-111 | 56.372 | 0.09 |
| STR048412 | TCCC | UTR5 | *PTGIR* | 12/0.95 | 204/0.00 | 18/0.02 | 12/0.95 | 13/0.72 | 15/0.27 | 15/0.27 | 15/0.27 | 11-27 | 13.953 | 0.08 |
| STR028748 | GAAA | upstream | *LINC02302* | 371/0.00 | 44/0.10 | 50/0.00 | 45/0.05 | 45/0.05 | 43/0.22 | 46/0.02 | 41/0.60 | 30-48 | 41.051 | 0.07 |
| STR003638 | GGGA | intronic | *JAK1* | 14/0.47 | 13/0.71 | 15/0.31 | 15/0.31 | 11/0.92 | 387/0.00 | 17/0.06 | 16/0.11 | 10-58 | 15.218 | 0.07 |

Supplementary Table 5. The characteristics of controls who contributed muscle tissue

| Patients | Control-1 | Control-2 | Control-3 |
| --- | --- | --- | --- |
| Sex | F | F | F |
| Age (years) | 64 | 69 | 65 |
| Disease for surgery | Lumbar Spinal Stenosis | Lumbar Spinal Stenosis | Lumbar Spinal Stenosis |


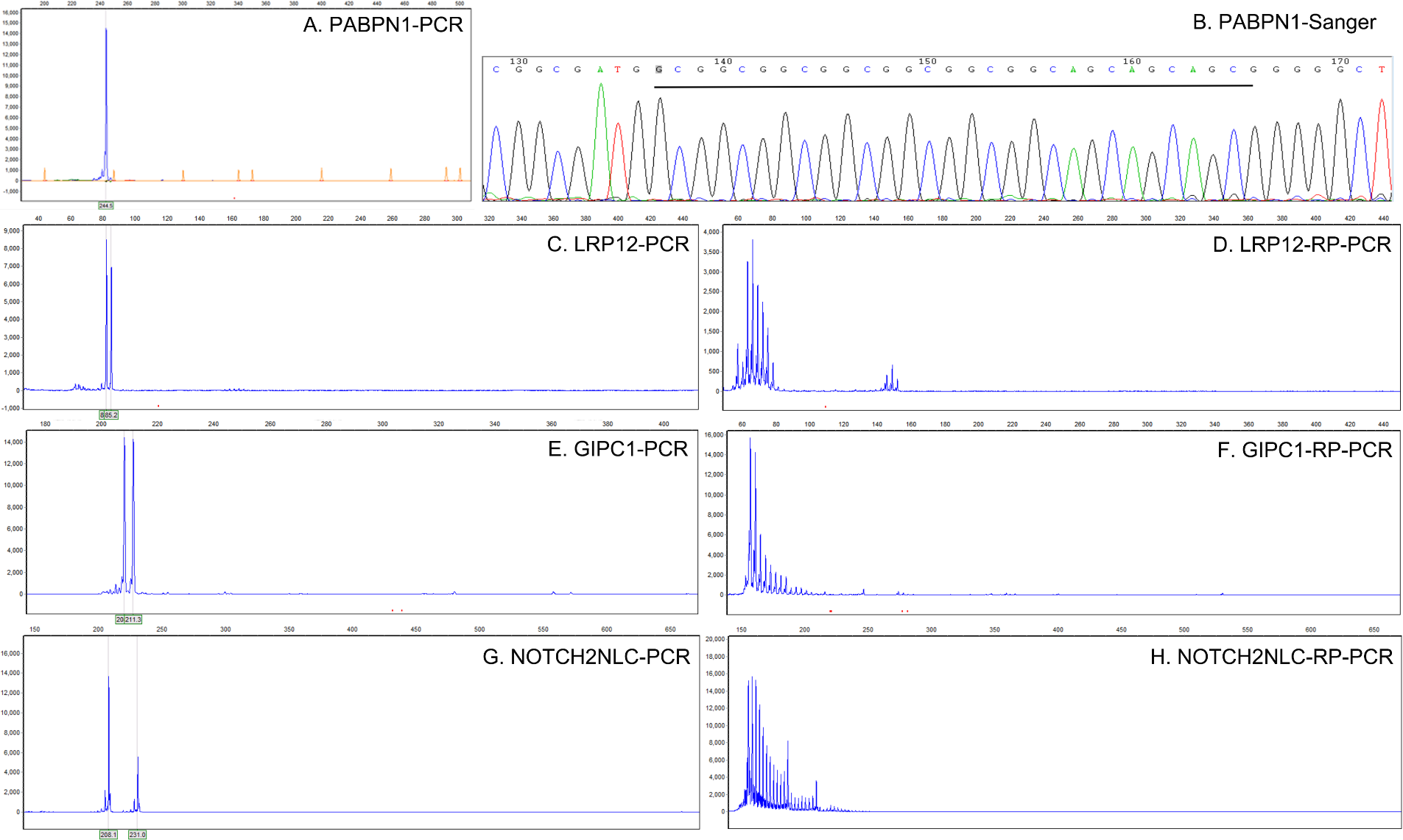
Supplementary Figure 1.

No repeat expansions in *PABPN1, LRP12*, *GIPC1* and *NOTCH2NLC* were detected in patient III-39. (A, B) No repeat expansions in *PABPN1* were detected with PCR and Sanger sequencing. (C, D) No repeat expansions in *LRP12* were detected by PCR and RP-PCR. (E, F) No repeat expansions in *GIPC1* were detected by PCR and RP-PCR. (G, H) No repeat expansions in *NOTCH2NLC* were detected by PCR and RP-PCR.


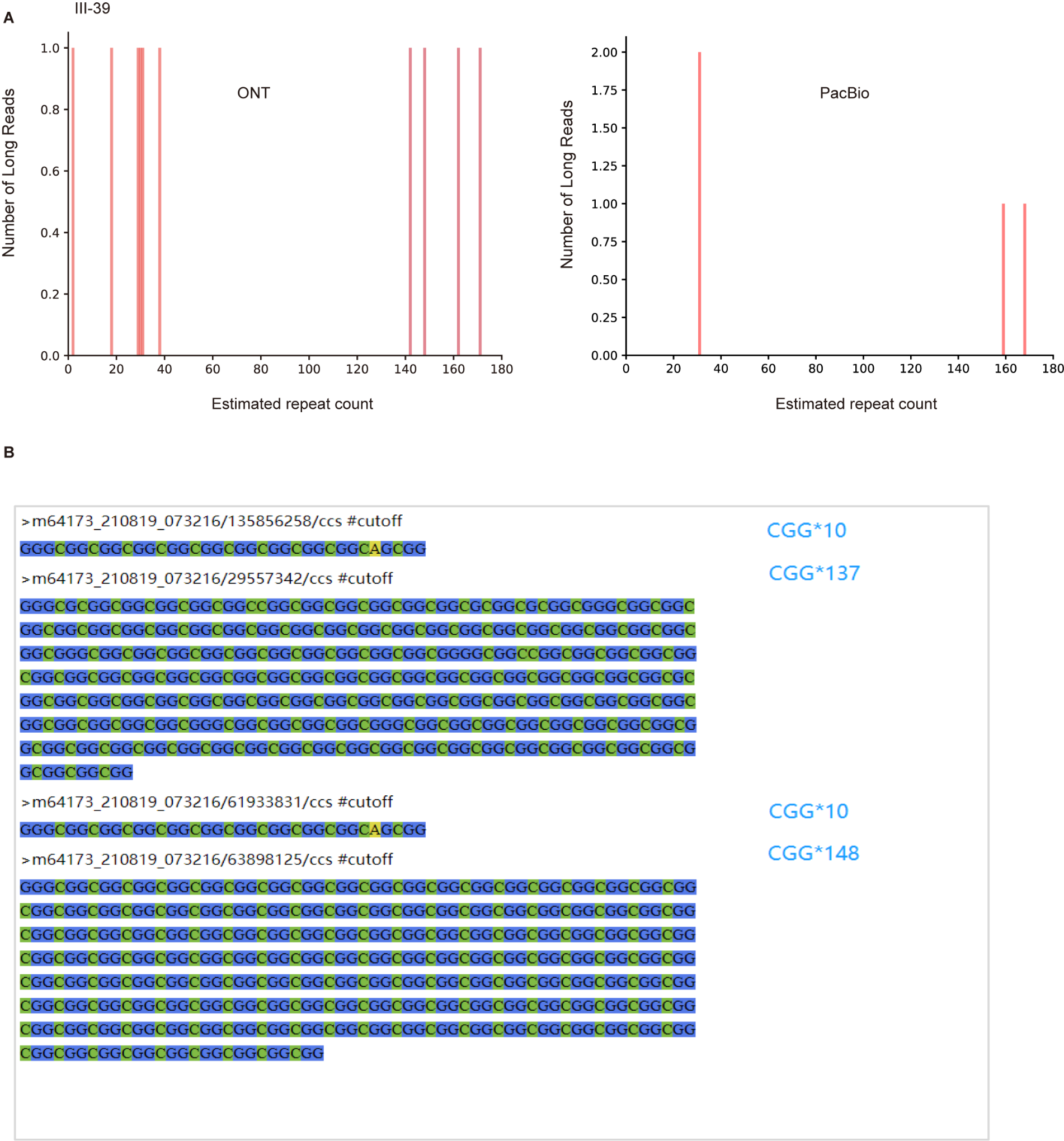
Supplementary Figure 2.

The CGG repeat expansion in the 5’UTR of *RILPL1* identified by PacBio CCS reads was consistent with ONT (A) and uninterrupted CGG repeat expansions while no other repeat form was detected (B).


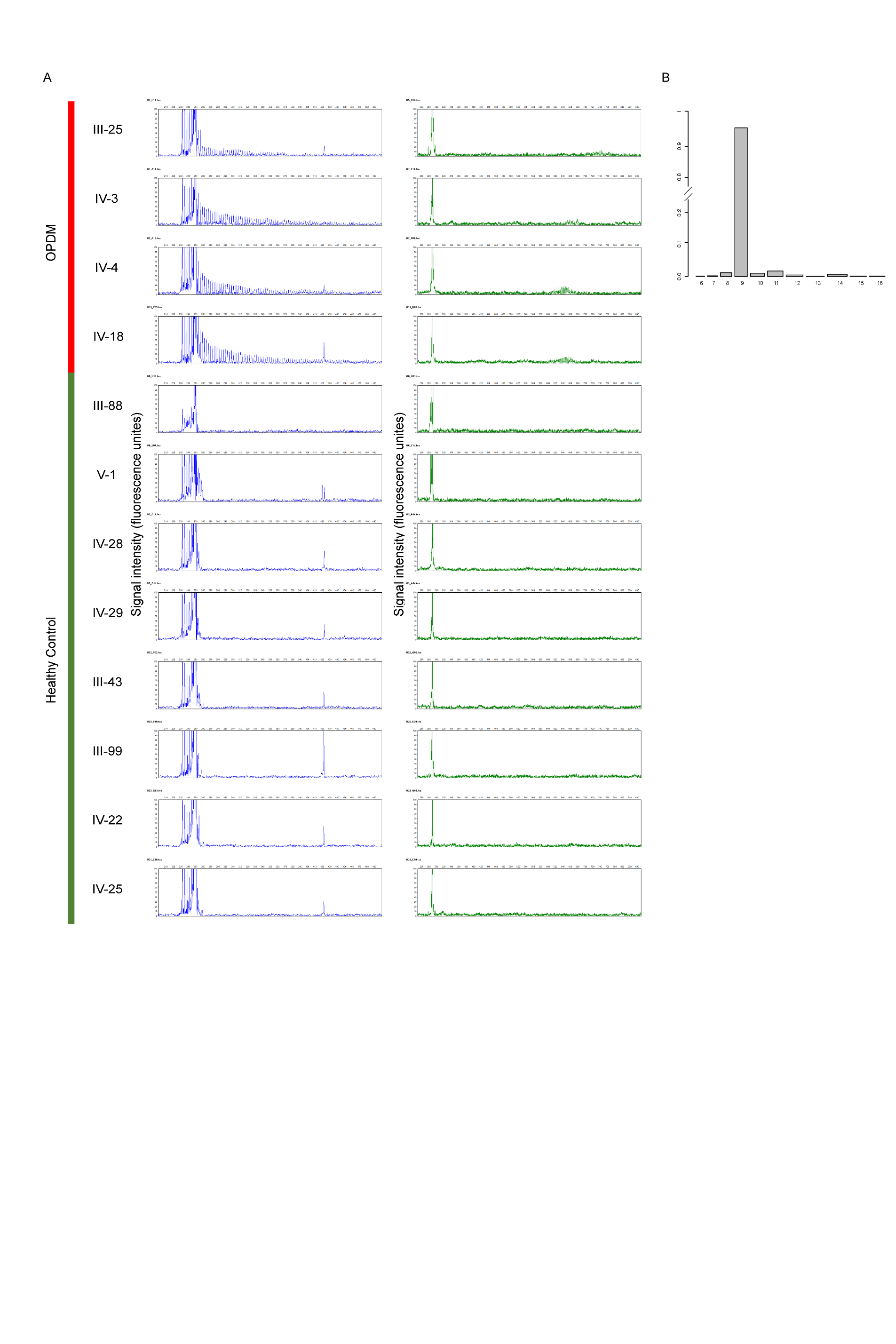
Supplementary Figure 3.

RP-PCR and AL-PCR analysis for additional family members (A) and frequency distribution of CGG repeat units in *RILPL1* among 1,000 healthy controls (B).


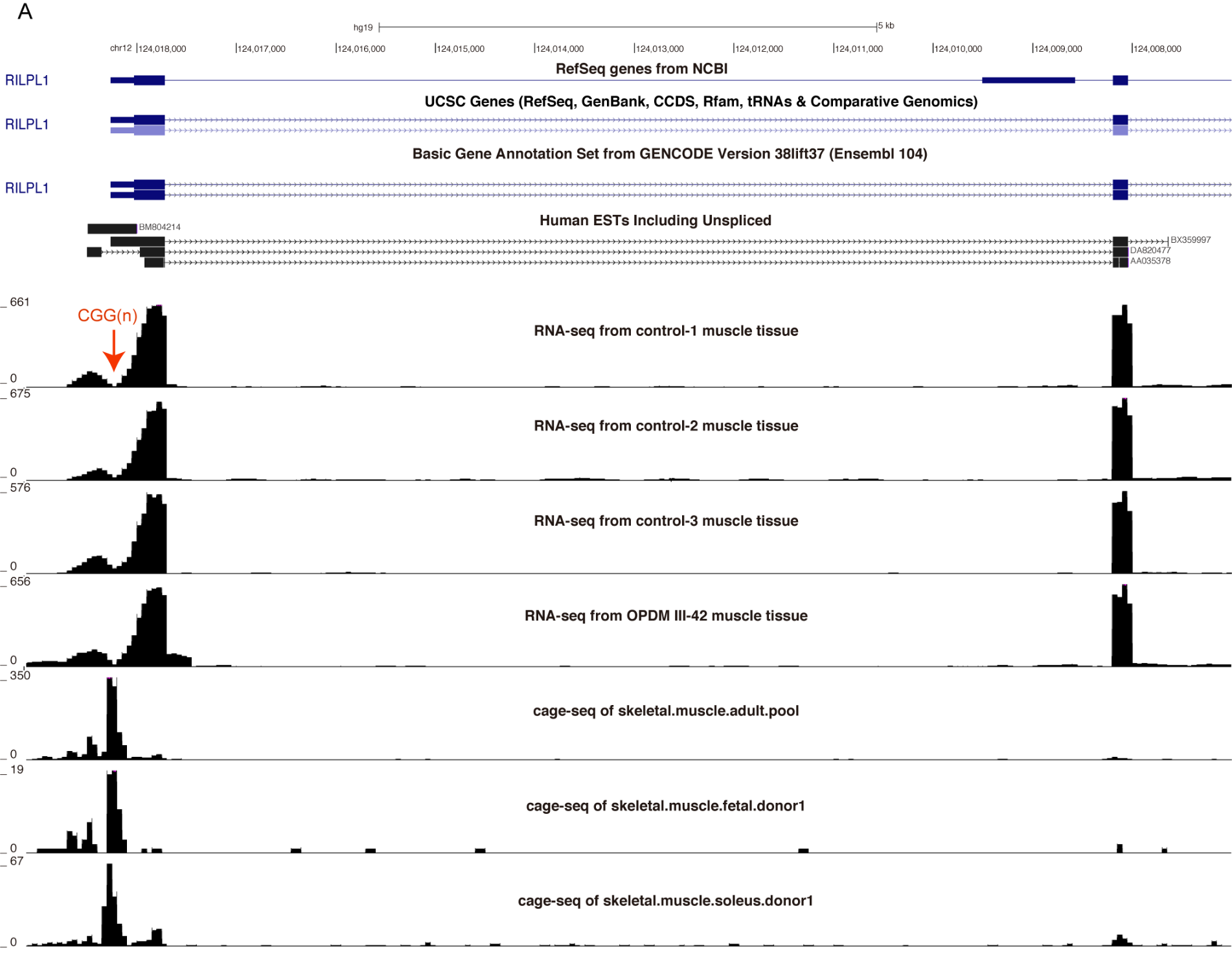
Supplementary Figure 4.

Gene annotation track from NCBI, UCSC, and GENCODE present traditional gene structures of *RILPL1*. While RNA ESTs, RNA-seq, and CAGE-seq provided evidences that *RIPL1* gene transcription might initiate from an upstream TSS and include expanded CGG repeats as part of its 5’UTR.


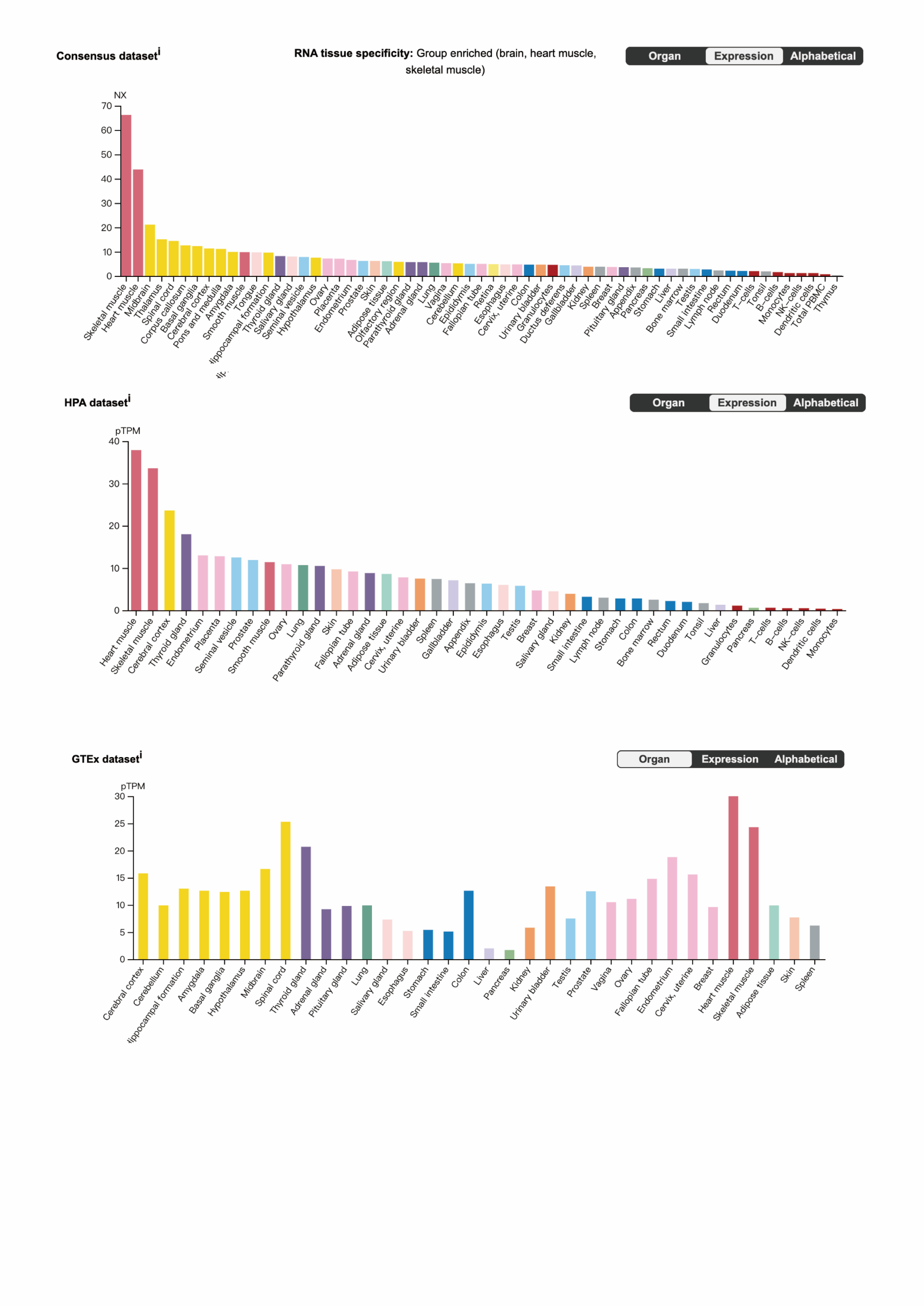
Supplementary Figure 5.

RNA expression level from Consensus dataset, HPA dataset, and GTEx dataset show that *RILPL1* is highly expressed in muscle and brain tissues.


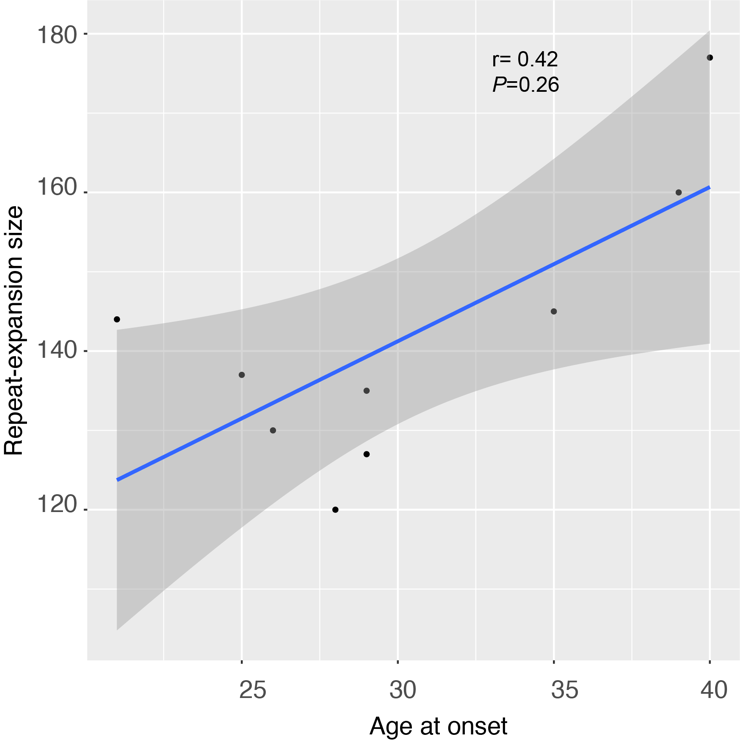


Supplementary Figure 6.

Correlation analysis between the number of CGG repeats and the age at onset in 9 OPDM4-affected individuals.
